# Immunofluorescence Targeting PBP2a Protein: A New Potential Methicillin Resistance Screening Test

**DOI:** 10.3389/fvets.2021.740934

**Published:** 2021-11-30

**Authors:** Serenella Silvestri, Elisa Rampacci, Valentina Stefanetti, Michele Trotta, Caterina Fani, Lucia Levorato, Chiara Brachelente, Fabrizio Passamonti

**Affiliations:** ^1^Department of Veterinary Medicine, University of Perugia, Perugia, Italy; ^2^CDVet Laboratorio Analisi Veterinarie, Rome, Italy; ^3^Department of Medicine and Surgery, University of Perugia, Perugia, Italy

**Keywords:** *Staphylococcus pseudintermedius*, methicillin resistance, fluorescent antibody technique, penicillin-binding protein 2a, antimicrobial drug resistance, pyoderma, dogs, humans

## Abstract

The indiscriminate use of first-line drugs contributed to the spread of resistant bacteria, a major concern for both human and veterinary medicine. Methicillin resistance is acquired through the *mecA* gene, which encodes for the PBP2a protein and lends the resistance to β-lactams. Verifying the correspondence between gene harboring and protein expression and accelerating methicillin resistance diagnosis is critical to improve the management of antimicrobial administration and to reduce the spread of drug resistances. We tested the applicability of immunofluorescence targeting PBP2a protein to identify a new potential methicillin resistance screening test, ancillary to conventional culture methods. We collected 26 clinical *Staphylococcus pseudintermedius* (SP) isolates: 25 from canine pyoderma and 1 from dermatitis in a dog owner. SP is one of the most important etiological agents in canine pyoderma and can harbor the *mecA* gene. We performed PCR for *mecA* gene detection, broth microdilution (BMD) for phenotypic methicillin resistance, and immunofluorescence targeting PBP2a protein. Compared to the PCR as the gold standard, immunofluorescence showed an apparent prevalence of 34.6% vs. a true prevalence of 53.8%, with 100% specificity, 64.3% sensitivity, and 80.8% diagnostic accuracy. PBP2a expression showed isolate-dependent variability: in some isolates, most of the bacterial cells showed an intense and clearly membranous pattern, while in others only a few of them could be detected. Performing the assay in duplicate improved the diagnostic accuracy. Since the *mecA* gene is shared among the members of the *Staphylococcus* genus, the test can be applied to identify methicillin resistance independently from the staphylococcal species, both in human and animal samples. Being a rapid and easy method and providing the unique possibility to study the expression of PBP2a by directly visualizing the morphology, it could represent a new interesting tool for both research and diagnostics. To accelerate methicillin resistance diagnosis, it would be worth further testing of its performance on cytological samples.

## Introduction

The indiscriminate use of first-line drugs has sparked off the development of resistance mechanisms to antimicrobials by bacteria over time. This is a growing problem afflicting both human and veterinary medicine, so that, in 2019 the World Health Organization (WHO) included the antimicrobial-resistance in the list of the ten major threats to human health (1, 2). The spreading worldwide of methicillin resistance in *Staphylococcus aureus* (SA) is a particular health concern, that poses serious problems in the choice of the proper therapy (2, 3). Methicillin resistance is due to the acquisition and expression of the *mecA* gene. It is located on a mobile element called staphylococcal cassette chromosome *mec* (SCC*mec*) (4) and can be easily transferred between staphylococcal species (5).

*Staphylococcus pseudintermedius* (SP) is a normal colonizer of the dog skin, which often acts as an opportunistic pathogen, and is one of the most important pyogenic agents in canine pyoderma. Failure in identifying/resolving the primary cause of pyoderma, inappropriate therapy, antimicrobial resistance, or lack of owner's compliance can lead to infection recurrence or persistence and repeated therapy (6, 7).

Similarly to SA, SP can acquire resistance to β-lactams, the most used antimicrobial drugs, through the *mecA* gene. The gene encodes for penicillin-binding protein 2a (PBP2a), resulting in an altered cell wall composition and lower affinity for β-lactams (4, 8).

In SA, a strain-dependent variability in methicillin/oxacillin resistance level is reported (4, 9). Additionally, despite the detection of the *mecA* gene, some isolates were found susceptible to oxacillin (OXA). This status was defined as the “pre-methicillin-resistant” phenotype (10, 11). These previous findings suggest that the *mecA* gene harboring could not correspond to the protein expression. Indeed, Rohde et al. underlined the importance of verifying the congruity between gene presence and the expression of the related protein (12). In experimental conditions, they demonstrated that an immunofluorescence test can be successfully employed for this purpose, suggesting its use as a rapid screening test for susceptibility. To the best of our knowledge, similar studies have never been conducted on clinical isolates of SP.

Additionally, SP isolates harboring the *mecA* gene are often resistant to other classes of antimicrobial agents, showing a “multi-drug resistant” (MDR) phenotype, which increases the effort of establishing an adequate targeted therapy (7, 13–15). SP also has a zoonotic potential and people in close contact with dogs (e.g., pet owners, veterinarians) are at maximum risk for infection, especially if they have a compromised immune system (6, 7, 16).

In such a context, speeding up the detection of methicillin resistance is a key factor to avoid choosing an inappropriate antimicrobial agent that would affect both the disease resolution and the further development of resistances (13, 17–19).

Our study aimed firstly to test the possibility to use a commercially available antibody targeting PBP2a protein in methicillin-resistant *Staphylococcus aureus* (MRSA), never validated in immunofluorescence or tested in methicillin-resistant *Staphylococcus pseudintermedius* (MRSP). Secondly, to evaluate the performance of immunofluorescence targeting PBP2a protein on clinical SP isolates from canine pyoderma. We compared those findings with the minimum inhibitory concentration (MIC) of OXA obtained by broth microdilution (BMD) and with PCR for the *mecA* gene, to investigate the agreement between the methods, as well as the matching between *mecA* gene harboring and PBP2a protein expression. Finally, we sought to explore the potentiality of this technique as a rapid screening test ancillary to conventional culture methods.

## Materials and Methods

### Bacteria Isolation and Antimicrobial Susceptibility Testing

Twenty-six SP isolates, previously included in a larger study on susceptibility testing methods comparison (20), were used in this study: 25 were isolated from canine pyoderma and 1 from a dermatitis sample of a dog owner. Only one isolate per subject was collected. Bacteria were isolated in clinical microbiology laboratories of Central Italy during routine work throughout 2019. Identification of the isolates was performed to the species level both by PCR-restriction fragment length polymorphism approach (RFLP), based on the detection of the MboI restriction site on *pta* locus (21), and by the Vitek-2 system (bioMérieux Inc., Durham, NC), using the most up-to-date GP ID cards, as previously described (20). Before testing, all isolates were cultured from −20°C storage onto Mannitol Salt Agar plates supplemented with 5% v/v Egg Yolk Emulsion and sub-cultured on cation-adjusted Mueller-Hinton agar (CAMHA). The MICs of OXA for the selected isolates, which is the recommended method to phenotypically predict methicillin resistance in SP, were investigated by BMD as previously described (20). Additionally, the MICs for amoxicillin/clavulanate, cephalothin, gentamicin, enrofloxacin, clindamycin, trimethoprim/sulfamethoxazole, doxycycline, and mupirocin were also determined (20). Bacteria resistant to at least three antimicrobial classes were classified as MDR (22). A methicillin-resistant SP isolate, from which the *mecA* gene was sequenced, was used as a positive control in PCR and immunofluorescence assay.

### PCR-Based Identification of *mecA* Gene

PCR for the *mecA* gene is the gold standard method for the detection of methicillin resistance (23). DNA from pure SP cultures was extracted by boiling. Bacterial colonies were resuspended in 500 μL of ultrapure molecular biology-grade water and subjected to boiling at 100°C. The suspension was cooled on ice and centrifuged at 14,000 rpm for 10 min. The supernatants were collected for conventional PCR analyses. Single PCR amplifications were performed in 25-μL reaction mixtures using Recombinant Taq DNA polymerase (Takara, Dalian, China) according to the manufacturer's instructions. The chromosomic *mecA* gene was amplified using 0.4 μM of primer f-AAAATCGATGGTAAAGGTTGGC and r-AGTTCTGCAGTACCGGATTTGC (Sigma–Genosys, Milan, Italy) (24). All PCR were performed in the GeneAmp PCR System 2400 thermocycler (Applied Biosystems, Foster City, CA), according to the following amplification conditions: initial denaturation at 94°C for 5 min, followed by 40 cycles of amplification at 94°C for 30 s, annealing at 55°C for 30 s, extension at 72°C for 1 min, and a final extension step at 72°C for 5 min. Positive control, from which the *mecA* gene was previously sequenced, a negative control (negative sample), as well as a negative reaction mix control (containing the reagents and water instead of DNA), were included in each run. The presence and size of the amplified products were confirmed by electrophoresis on 1.5% agarose gel.

### Immunofluorescence

For bacteria fixation, a modified protocol was used (12). Briefly, all isolates were fixed adding 3 volumes of 4% formaldehyde in Tris-buffered saline (TBS) buffer. After 1 h incubation at 4°C, bacteria were washed 3 times through centrifugation and resuspension in TBS buffer. Finally, bacteria were suspended in a 1:1 ethanol/TBS solution and used directly. Bacteria solution could be also stored at −20°C before use. Ten μl of each bacteria solution were pipetted on a glass slide and dried for 3 min at 52°C on a hot plate. Slides were stored in the dark until used.

To permeabilize bacteria, slides were incubated with a lysozyme solution (213 μg/ml in TRIS buffer 50 mM, pH 7; Lysozyme, 8259.1, Carl Roth, Karlsruhe, Germany) for 30 min at room temperature (RT) in a humidified chamber and rinsed in TBS buffer. Slides were blocked with blocking buffer (2% bovine serum albumin in TBS buffer; bovine serum albumin solution, A7034, Sigma-Aldrich, Saint Louis, MO) for 10 min at RT. Since the *mecA* gene is shared by the *Staphylococcus* genus (5, 25), we used a specific anti-PBP2a primary antibody validated for application in ELISA and WB to detect MRSA (130-10073, RayBiotech, Peachtree Corners, GA), thus testing its applicability in immunofluorescence and in the detection of MRSP. Slides were incubated overnight at 4°C in a humidified chamber with the rabbit primary antibody diluted 1:200 in blocking buffer. Slides were rinsed in TBS and incubated with a biotinylated goat anti-rabbit secondary antibody (BA-1000, Vector Laboratories, Burlingame, CA) diluted 1:200 in TBS for 1 h at RT. After rinsing in TBS buffer, samples were incubated in a dark humidified chamber with the Alexa Fluor^®^ 488 streptavidin conjugate (S-32354, Life Technologies, Paisley, UK) diluted 1:200 in blocking buffer for 1 h at RT. Finally, the rinsed slides were incubated in the dark with 4', 6-diamidino-2-phenylindole, dilactate (DAPI; D3571, Invitrogen, Eugene, OR) diluted 1:1000 in TBS buffer for 5 min at RT. After carefully rinsing in TBS, slides were coverslipped with ProLong^TM^ Gold antifade mountant (P36930, TermoFisher Scientific, Rockford, USA). As a positive control for the immunofluorescence assay, we used the isolate used for PCR validation, which resulted as methicillin-resistant also by BMD. The same isolate was used as a negative control, omitting the primary antibody. To verify the specificity of the antibody, we selected one of the isolates confirmed for being methicillin-sensitive both by PCR and BMD as additional negative control (Table 1).

**Table 1:** Results of methicillin resistance investigation in the SP isolates and their MDR status.

**Isolate**	MIC*	**Category**	**PCR**	IF**	**MDR**
Pos ctr	>32	R	+	+	+
Neg ctr	≤ 0.125	S	−	−	−
SP01	≤ 0.125	S	−	−	−
SP02	≤ 0.125	S	−	−	−
SP03	≤ 0.125	S	−	−	−
SP04	>32	R	+	+	+
SP05	>32	R	+	+	+
SP06	>32	R	+	+	+
SP07	>32	R	+	+	+
SP08	≤ 0.125	S	−	−	−
SP09	8	R	+	+	+
SP10	≤ 0.125	S	−	−	−
SP11	1	R	−	−	+
SP12	>32	R	+	+	+
SP13	>32	R	+	−	+
SP14	1	R	+	−	−
SP15	0.25	S	−	−	−
SP16	≤ 0.125	S	−	−	−
SP17	>32	R	+	+	+
SP18	≤ 0.125	S	−	−	−
SP19	>32	R	+	−	+
SP20	≤ 0.125	S	−	−	−
SP21	>32	R	+	+	+
SP22	0.5	R	+	−	−
SP23	1	R	+	−	ND
SP24	≤ 0.125	S	−	−	−

*R, resistant; S, sensitive; ND, not determined; SP24, human isolate*.

* *Results of BMD were previously published (15)*.

** *The immunofluorescence (IF) results after 2 replicates are shown*.

We performed two technical replicates of the immunofluorescence assay. Except for the positive and negative controls, all the slides were blindly evaluated for PBP2a expression by one operator to avoid inter-operator variability. When at least one of the immunofluorescence assays was positive, we considered the PBP2a expressed. Samples were evaluated using a fluorescent microscope Olympus BX51 equipped with the camera Nikon mod.DS-Qi2Mc. NIS-ELEMENTS D software was used for image analysis.

### Statistical Analysis

For descriptive statistics data are shown as absolute and relative frequencies. To evaluate the inter-method agreement, we calculated both the categorical agreement and Cohen's kappa. The categorical agreement is represented by the proportion of the isolates producing the same category result (methicillin-sensitive or -resistant) as compared to the reference method. Major error (ME) was reported when the reference test returned a sensitive result, while the method under evaluation returned resistant. Conversely, a very major error (VME) indicates that the reference test returned a resistant result but the method under evaluation returned sensitive (26). Unweighted Cohen's kappa with 95% confidence interval (CI_95%_) based on bootstrap (10,000 replicates) was calculated and interpreted as previously described (27). Finally, referring to PCR as the gold standard, the sensitivity, specificity, positive predictive value (PPV), negative predictive value (NPV), likelihood ratio for a positive test, likelihood ratio for a negative test, and diagnostic accuracy of the immunofluorescence assay were also calculated according to previous literature (28, 29). Statistical analyses were performed using the software R (R version 4.0.3) (30).

## Results

**Figure 1:**
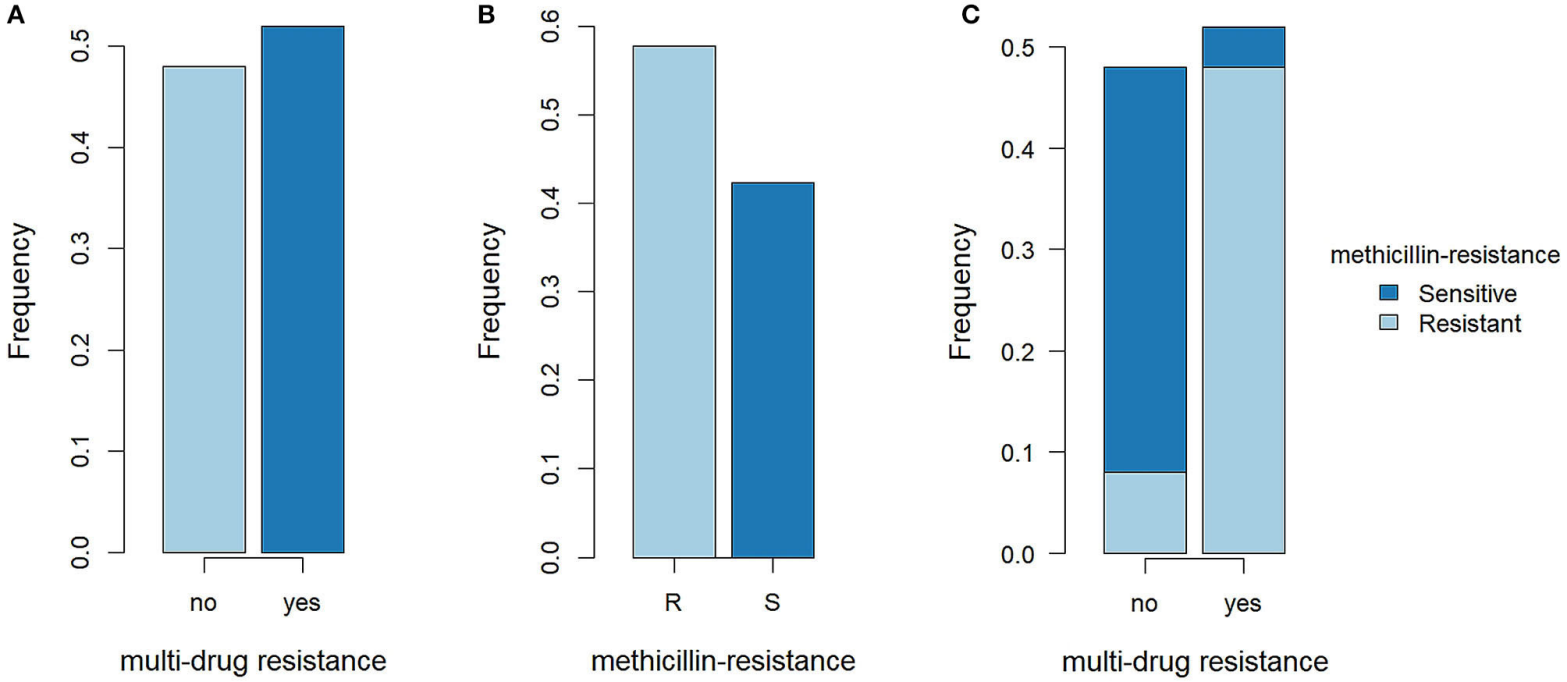
Methicillin resistance and multi-drug resistance in SP isolates. **(A)** Proportions of multidrug-resistant (MDR) SP and not MDR SP. R, resistant; S, sensitive. **(B)** Proportions of methicillin-resistant SP (MRSP) and methicillin-sensitive SP (MSSP). **(C)** Proportions of MRSP and MSSP in MDR SP and not MDR SP.

We examined 25 SP isolates from canine pyoderma and 1 from a dermatitis sample of a dog owner (Table 1). Based on MIC determination, 57.7% (15/26) of the samples were resistant to OXA and 52% (13/25; one case not determined) were MDR. The majority of MDR SP were OXA resistant (92.3%, 12/13; Figure 1).

Based on the results of PCR, 14/26 (53.8%) SP isolates harbored the *mecA* gene, and, with only one exception, results of MIC evaluation and PCR were in agreement. Specifically, the isolate SP11 was classified as resistant to OXA with a MIC of 1 mg/L, but PCR did not detect the *mecA* gene for this SP. Consequently, one ME was produced and the categorical agreement between the two methods was 96.1% (25/26).

Overall, a clear division between OXA MICs was found between *mecA*-positive and -negative isolates. The majority of *mecA*-positive SP (10/14, 71.4%) showed an OXA MIC > 32 mg/L while, except for SP11, all *mecA*-negative isolates had an OXA MIC ≤ 0.25 mg/L (11/12, 91.6%; Supplementary Figure 1A). A low level of resistance to OXA was found also for the isolates SP14 and SP21, but, in these cases, PCR detected the *mecA* gene (Table 1).

**Figure 2:**
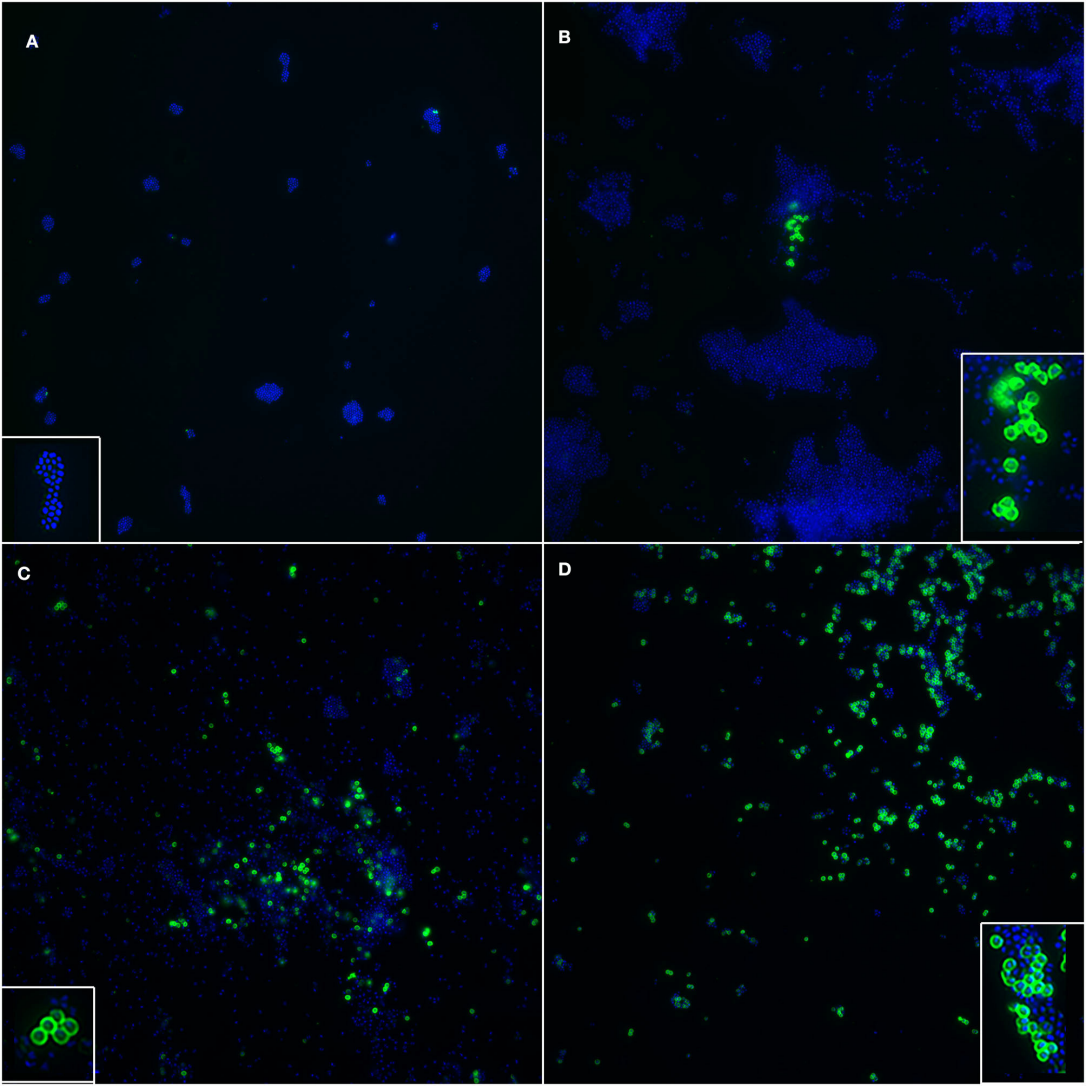
Immunofluorescence targeting PBP2a protein in SP, showing isolate-dependent variability in PBP2a expression level. **(A)** Negative control. Insert: a cluster of SP whose cell walls stained negative and only the nucleoid can be seen (blue). **(B)** A cluster of SP clearly expressing PBP2a protein (green) is shown; the majority of SP does not express the protein. Insert: a magnification of cell walls staining positive, with a well-defined membranous pattern. **(C)** Several SP, both in clusters and sparse, stained positive, while a large proportion of them is negative. Insert: bacteria with cell wall expression of PBP2a protein. **(D)** Most of the SP showed positive cell walls. Insert: a cluster of bacteria where most of them have distinct positivity of the cell walls, together with other negative bacteria where only the nucleoid is stained. Blue: DAPI; Green: Alexa Fluor^®^ 488.

**Figure 3:**
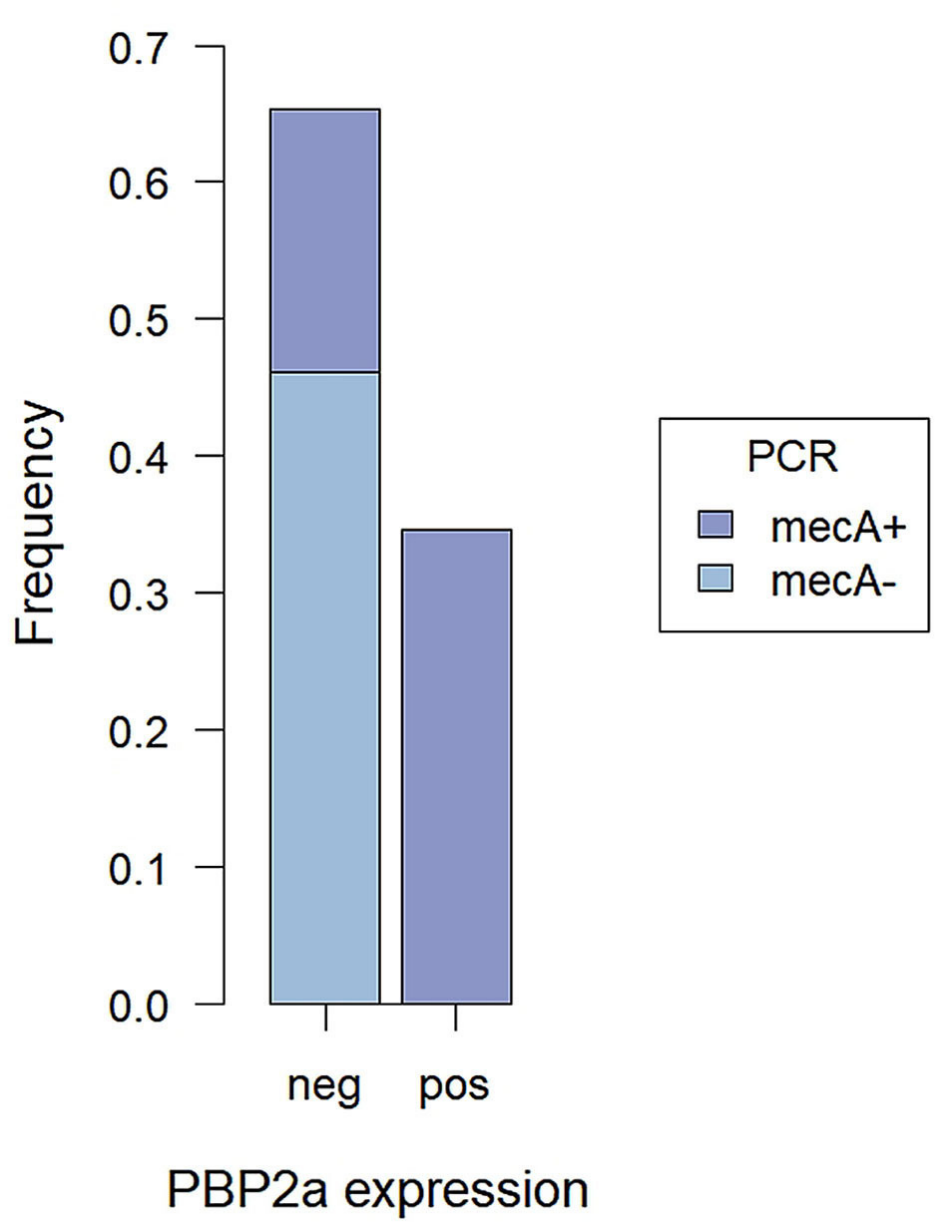
Proportions of *mecA*+ and *mecA*− SP tested by PCR among cases with detected and not PBP2a expression by immunofluorescence assay.

In the immunofluorescence assay, the PBP2a expression has a clear membranous pattern with isolate-dependent variability: while in some isolates the expression was evident in most of the bacteria on the slide, in others PBP2a was expressed by a minor proportion of SP, sometimes making the detection challenging (Figure 2 and Supplementary Table 1). The agreement between the two immunofluorescence replicates was almost perfect (*k* = 0.82, CI_95%_ = 0.52–1.00), since only for 2/26 (7.7%) isolates the results disagreed. In both cases, the positive bacteria identified on the slides were scarce. However, when at least one of the two replicates showed detectable PBP2a protein, the isolate was classified as positive. Overall, 9/26 isolates resulted positive (Table 1), with an apparent prevalence of 34.6% vs. a true prevalence of 53.8% (based on PCR results; Table 2). Indeed, although the agreement with PCR was substantial (*k* = 0.62, CI_95%_ = 0.34–0.91), in 5/26 (19.2%) isolates PBP2a was not detected while PCR demonstrated *mecA* gene harboring (Figure 3). The isolate SP11, where PCR and MIC showed opposite results, was correctly classified as negative for PBP2a expression through both immunofluorescence replicates. Consequently, the categorical agreement with PCR for the *mecA* gene was 80.8% (21/26), while with OXA MIC was 76.9% (20/26). Particularly, in 3/5 (60%) cases in which PBP2a expression was not evident by immunofluorescence, the MICs ranged between 0.5 and 1 mg/L (Supplementary Figure 1B), while when it was ≥8 mg/L PBP2a expression was generally detected (9/11, 81.8%).

**Table 2:** Measures of diagnostic test accuracy.

**Measure**	**Estimate**	**CI_95%_**
Apparent prevalence	34.6%	17.2–55.7%
True prevalence	53.8%	33.4–73.4%
Sensitivity	64.3%	35.1–87.2%
Specificity	100%	64.0–100%
Positive predictive value	100%	55.5–100%
Negative predictive value	70.6%	44.0–89.7%
Likelyhood ratio for positive test	inf	NA
Likelyhood ratio for negative test	0.357	0.177–0.721
Diagnostic accuracy	80.8%	60.6–93.4%

*CI_95%_, 95% confidence interval*.

*inf, infinity; NA, non applicable*.

Since no false-positive results were obtained, both the specificity and the positive predictive value (PPV) reached 100%, while the sensitivity and the negative predictive value (NPV) were lower. Overall, the diagnostic accuracy of the method was 80.8% (CI_95%_ = 60.6–93.4%; Table 2).

Finally, in our case series, all the SP isolates showing positivity by immunofluorescence were MDR bacteria.

## Discussion

Pyoderma is a common skin problem in the canine species and frequently leads to antimicrobial use in clinical practice (7). Since the spreading of resistant bacteria is growing and therapeutic options are limited, antimicrobial management optimization is crucial (1, 2, 31). SP, one of the most important etiological agents involved in canine pyoderma (32), can harbor the *mecA* gene that, coding for the PBP2a protein, mediates the methicillin resistance (4).

We tested a new technique potentially applicable in diagnostics as a rapid screening test to detect PBP2a expression. This would help to identify methicillin-resistant staphylococci, providing the clinician with an initial guide for starting the therapy while waiting for antimicrobial susceptibility test results.

Comparing the immunofluorescence assay to PCR for the *mecA* gene, the gold standard for identification of MRSP, the agreement was substantial, but in 19.2% of cases it failed to detect MRSP. Given the specificity of the antibody chosen, we had no false-positive results and the specificity was 100%. However, the sensitivity of the assay was much lower, being equal to 64.3%. Often, the lack of detection of methicillin resistance involved the isolates with low MICs (0.5–1 mg/L). This could be due to the lower sensitivity of immunofluorescence compared to PCR. Additionally, an isolate-dependent variability in PBP2a expression level was observed in our study. When the positive bacteria on the slide are a few, they could be missed, resulting in false-negative results. In our case series, in 2 methicillin-resistant SP isolates, one of the replicates failed to detect the expression of the PBP2a protein. Hence, repeating the assay in duplicate can improve the diagnostic accuracy. Additionally, *mecA* gene expression can be induced by OXA and cefoxitin stimulation (23, 33), but the isolates used in our study for immunofluorescence were not previously exposed to antimicrobials, in order to mimic diagnostic conditions. As a result, the PBP2a protein could have a lower or no expression in some isolates, affecting the general sensitivity of the method. Finally, the sample size we used was relatively limited, hence the lacking of PBP2a detection in a few samples might be overweighed. Testing the method on a larger number of samples might help obtain a more precise evaluation of its performance.

Whit one exception, the results of MIC and PCR overlapped in all cases. The mechanism of OXA resistance, in this case, remains to be determined. Notably, immunofluorescence classification of this isolate was in agreement with PCR, which is why the categorical agreement with BMD was lower compared to those with PCR.

Despite its limitations, immunofluorescence has several advantages. In our study, the diagnostic accuracy reached 80.8%, showing high reliability when methicillin-resistant SP are identified (PPV = 100%; NPV = 70.6%). In agreement with previous studies reporting a high prevalence of MDR among MRSP (14, 34), in our case series, all of the isolates expressing PBP2a protein were also MDR. Accordingly, the detection of PBP2a expression could help suspect MDR. Results of immunofluorescence targeting PBP2a protein can be rapidly obtained, especially if the primary antibody incubation time is shortened. The method might also be easily applied on cytological samples with the potential to get the results within the same day, so it would be worth testing it in this application. As a further development, a modified method could be employed in immunohistochemistry, allowing the study of the resistant bacteria directly on tissue samples and representing a new interesting tool for both research and diagnostics. However, the suitability of the selected antibody in immunohistochemistry has to be determined. Moreover, since the *mecA* gene is shared by several staphylococci, including SA (5, 25), the methicillin resistance could be detected independently from the staphylococcal species isolated in both human and animal hosts.

To the best of the author's knowledge, two commercially available kits can be used for the rapid detection of PBP2a protein on cultured colonies. The Alere PBP2a Culture Colony Test is a sensitive and specific immunochromatographic assay to test isolates. Although the test itself is very simple and rapid, the colonies should be cultured for at least 24 h and the highest sensitivity is reached when bacteria are harvested from the edges of the cefoxitin zone of growth inhibition (23, 25). The other is the PBP2a latex agglutination test, whose sensitivity and specificity are almost comparable to PCR (35, 36). However, it is technically more complicated and needs additional equipment (3). Being performed on isolates, these commercial tests strictly depend on the timing of bacteria growth. Compared to those tests, the immunofluorescence assay has a lower sensitivity but a corresponding specificity. It is a simple technique and can be carried on in pathology laboratories that routinely perform immunocytochemistry or immunohistochemistry (ICC/IHC) and are equipped with a fluorescent microscope. It is rapid and, if applied on cytological samples, it might be carried out independently from bacteriological culture. Finally, it provides the unparalleled possibility to study the expression of PBP2a by directly visualizing the morphology, opening new possibilities for research purposes.

In conclusion, we demonstrated that immunofluorescence can be successfully used to detect the PBP2a protein in SP isolates, hence methicillin-resistant bacteria. When compared to the gold standard method (PCR for *mecA* gene), immunofluorescence targeting PBP2a protein showed good diagnostic accuracy, with 100% specificity, although the sensitivity is lower. It is a rapid and easy method that can represent a new interesting tool for both research and diagnostics. It would be worth testing its performance on cytological samples to further accelerate the diagnosis of methicillin resistance in SP.

## Data Availability Statement

The original contributions presented in the study are included in the article/Supplementary Material, further inquiries can be directed to the corresponding author/s.

## Author Contributions

SS: conceptualization, design, investigation, first draft writing, data acquisition, data analysis, visualization, and software. ER: conceptualization, design, investigation, first draft sections writing, data acquisition, data analysis, and software. VS: conceptualization, investigation, data acquisition, and software. MT and LL: investigation, data acquisition, and software. CF: funding acquisition, resources, and supervision. CB: conceptualization, design, resources, and supervision. FP: conceptualization, funding acquisition, resources, and supervision. All authors contributed to the manuscript's critical revision and editing and approved the submitted version.

## Funding

This research was funded by CDVet Laboratorio Analisi Veterinarie (Roma, Italy).

## Conflict of Interest

The authors declare that the research was conducted in the absence of any commercial or financial relationships that could be construed as a potential conflict of interest.

## Publisher's Note

All claims expressed in this article are solely those of the authors and do not necessarily represent those of their affiliated organizations, or those of the publisher, the editors and the reviewers. Any product that may be evaluated in this article, or claim that may be made by its manufacturer, is not guaranteed or endorsed by the publisher.

## References

[1] World Health Organization (WHO) I. Ten *Treats to Global Health in 2019*. (2019). Available online at: https://www.who.int/news-room/spotlight/ten-threats-to-global-health-in-2019

[2] Yarbrough M Lainhart W Burnham C-A. Epidemiology, clinical characteristics, antimicrobial susceptibility profiles of human clinical isolates of Staphylococcus intermedius group. J Clin Microbiol. (2018) 56:e01788–17. 10.1128/JCM.01788-17 29305548 PMC5824035

[3] Yamada K Wanchun J Ohkura T Murai A Hayakawa R Kinoshita K. Detection of methicillin-resistant *Staphylococcus aureus* using a specific anti-PBP2a chicken IgY antibody. Jpn J Infect Dis. (2013) 66:103–8. 10.7883/yoken.66.103 23514905

[4] Ballhausen B Kriegeskorte A Schleimer N Peters G Becker K. The mecA homolog mecC confers resistance against β-lactams in *Staphylococcus aureus* irrespective of the genetic strain background. Antimicrob Agents Chemother. (2014) 58:3791–8. 10.1128/AAC.02731-13 24752255 PMC4068569

[5] Somayaji R Priyantha MAR Rubin JE Church D. Human infections due to *Staphylococcus pseudintermedius*, an emerging zoonosis of canine origin: report of 24 cases. Diagn Microbiol Infect Dis. (2016) 85:471–6. 10.1016/j.diagmicrobio.2016.05.008 27241371

[6] Bajwa J. Canine superficial pyoderma and therapeutic considerations. Diagnostic Dermatology. (2016) 52:204–6. 26834275 PMC4713004

[7] Loeffler A Lloyd D. What has changed in canine pyoderma. A narrative review. Vet J. (2018) 235:73–82. 10.1016/j.tvjl.2018.04.002 29704943

[8] Priyantha R Gaunt MC Rubin JE. Antimicrobial susceptibility of *Staphylococcus pseudintermedius* colonizing healthy dogs in Saskatoon, Canada. Can Vet J La Rev Vet Can. (2016) 57:65–9. 26740701 PMC4677612

[9] Pardos de la Gandara M Borges V Chung M Milheiriço C Gomes JP de Lencastre H. Genetic determinants of high-level oxacillin resistance in methicillin-resistant *Staphylococcus aureus*. Antimicrob Agents Chemother. (2018) 62:e00206–18. 10.1128/AAC.01096-18 29555636 PMC5971597

[10] Kuwahara-Arai K Kondo N Hori S Tateda-Suzuki E Hiramatsu K. Suppression of methicillin resistance in a mecA-containing pre-methicillin-resistant Staphylococcus aureus strain is caused by the mecI-mediated repression of PBP 2 production. Antimicrob Agents Chemother. (1996) 40:2680–5. 10.1128/AAC.40.12.2680 9124822 PMC163603

[11] Oliveira DC de Lencastre H. Methicillin-resistance in *Staphylococcus aureus* is not affected by the overexpression in trans of the mecA gene repressor: a surprising observation. Van Melderen L editor. PLoS ONE. (2011) 6:e23287. 10.1371/journal.pone.0023287 21829724 PMC3149077

[12] Rohde A Hammerl JA Al Dahouk S. Rapid screening for antibiotic resistance elements on the RNA transcript, protein and enzymatic activity level. Ann Clin Microbiol Antimicrob. (2016) 15:55. 10.1186/s12941-016-0167-8 27663856 PMC5035493

[13] Perreten V Kadlec K Schwarz S Andrsson UG Finn M Greko C. Clonal spread of methicillin-resistant *Staphylococcus pseudintermedius* in Europe and North America: an international multicentre study. J Antimicrob Chemother. (2010) 65:1145–54. 10.1093/jac/dkq078 20348087

[14] Stefanetti V Bietta A Pascucci L Marenzoni ML Coletti M Franciosini MP. Investigation of the antibiotic resistance and biofilm formation of *Staphylococcus pseudintermedius* strains isolated from canine pyoderma. Vet Ital. (2017) 53:289–96. 10.12834/VetIt.465.2275.6 29307122

[15] Ventrella G Moodley A Grandolfo E Parisi A Corrente M Buonavoglia D. Frequency, antimicrobial susceptibility and clonal distribution of methicillin-resistant *Staphylococcus pseudintermedius* in canine clinical samples submitted to a veterinary diagnostic laboratory in Italy: A 3-year retrospective investigation. Vet Microbiol. (2017) 211:103–6. 10.1016/j.vetmic.2017.09.015 29102103

[16] Phumthanakorn N Schwendener S Donà V Chanchaithong P Perreten V Prapasarakul N. Genomic insights into methicillin-resistant *Staphylococcus pseudintermedius* isolates from dogs and humans of the same sequence types reveals diversity in prophages and pathogenicity islands. PLoS ONE. (2021) 16:e0254382. 10.1371/journal.pone.0254382 34292970 PMC8297860

[17] Epstein CR Yam WC Peiris JSM Epstein RJ. Methicillin-resistant commensal staphylococci in healthy dogs as a potential zoonotic reservoir for community-acquired antibiotic resistance. Infect Genet Evol. (2009) 9:283–5. 10.1016/j.meegid.2008.11.003 19073283

[18] Vanderhaeghen W Van De Velde E Crombé F Polis I Hermans K Haesebrouck F. Screening for methicillin-resistant staphylococci in dogs admitted to a veterinary teaching hospital. Res Vet Sci. (2012) 93:133–6. 10.1016/j.rvsc.2011.06.017 21726884

[19] Kjellman EE Slettemeås JS Small H Sunde M. Methicillin-resistant *Staphylococcus pseudintermedius* (MRSP) from healthy dogs in Norway - occurrence, genotypes and comparison to clinical MRSP. Microbiologyopen. (2015) 4:857–66. 10.1002/mbo3.258 26423808 PMC4694142

[20] Rampacci E Trotta M Fani C Silvestri S Stefanetti V Brachelente C. Comparative performances of Vitek-2, disk diffusion, and broth microdilution for antimicrobial susceptibility testing of canine *Staphylococcus pseudintermedius*. J Clin Microbiol. (2021) 59:e0034921. 10.1128/JCM.00349-21 34132581 PMC8373018

[21] Bannoehr J Franco A Iurescia M Battisti A Fitzgerald JR. Molecular diagnostic identification of *Staphylococcus pseudintermedius*. J Clin Microbiol. (2009) 47:469–71. 10.1128/JCM.01915-08 19091817 PMC2643665

[22] Magiorakos A-P Srinivasan A Carey R Carmeli Y Falagas M Giske C. Multidrug-resistant, extensively drug-resistant and pandrug-resistant bacteria: an international expert proposal for interim standard definitions for acquired resistance. Clin Microbiol Infect. (2012) 18:268–81. 10.1111/j.1469-0691.2011.03570.x 21793988

[23] Wu M Burnham C Westblade L Dien Bard J Lawhon S Wallace M. Evaluation of oxacillin and cefoxitin disk and MIC breakpoints for prediction of methicillin resistance in human and veterinary isolates of Staphylococcus intermedius Group. Richter SS editor. J Clin Microbiol. (2016) 54:535–42. 10.1128/JCM.02864-15 26607988 PMC4767974

[24] Strommenger B Kettlitz C Werner G Witte W. Multiplex PCR assay for simultaneous detection of nine clinically relevant antibiotic resistance genes in *Staphylococcus aureus*. J Clin Microbiol. (2003) 41:4089–94. 10.1128/JCM.41.9.4089-4094.2003 12958230 PMC193808

[25] Arnold A Burnham C-A Ford B Lawhon S McAllister S Lonsway D. Evaluation of an immunochromatographic assay for rapid detection of penicillin-binding protein 2a in human and animal Staphylococcus intermedius Group, *Staphylococcus lugdunensi*s, and *Staphylococcus schleiferi* clinical isolates. J Clin Microbiol. (2016) 54:745–8. 10.1128/JCM.02869-15 26677248 PMC4767977

[26] Humphries Romney M Ambler J Mitchell SL Castanheira M Dingle T Hindler JA. CLSI methods development and standardization working group best practices for evaluation of antimicrobial susceptibility tests. J Clin Microbiol. (2018) 56:e01934–17. 10.1128/JCM.01934-17 29367292 PMC5869819

[27] Landis JR Koch GG. The measurement of observer agreement for categorical data. Biometrics. (1977) 33:159–74. 10.2307/2529310 843571

[28] Šimundić A-M. Measures of diagnostic accuracy: basic definitions. EJIFCC. (2009) 19:203–11. 27683318 PMC4975285

[29] Shim SR Kim S-J Lee J. Diagnostic test accuracy: application and practice using R software. Epidemiol Health. (2019) 41:e2019007. 10.4178/epih.e2019007 30999739 PMC6545496

[30] R Core Team. R: A Language and Environment for Statistical Computing. Vienna: R Foundation for Statistical Computing (2020).

[31] Committee for Medicinal Products for Veterinary Use I. Reflection Paper on Meticillin-Resistant Staphylococcus pseudintermedius. London: European Medicine Agency (2010).

[32] Miller W Griffin C Campbell K. Muller&Kirk's Small Animal Dermatology. 7th ed. Saint Louis, MI: Elsevier (2013).

[33] Dupieux C Bouchiat C Larsen A Pichon B Holmes M Teale C. Detection of mecC-positive *Staphylococcus aureus*: what to expect from immunological tests targeting PBP2a? J Clin Microbiol. (2017) 55:1961–3. 10.1128/JCM.00068-17 28298453 PMC5442554

[34] Little SV Bryan LK Hillhouse AE Cohen ND Lawhon SD. Characterization of agr Groups of *Staphylococcus pseudintermedius* Isolates from Dogs in Texas. D'Orazio SEF editor. mSphere. (2019) 4:e00033–19. 10.1128/mSphere.00033-19 30918056 PMC6437270

[35] Cavassini M Wenger A Jaton K Blanc DS Bille J. Evaluation of MRSA-Screen, a simple anti-PBP 2a slide latex agglutination kit, for rapid detection of methicillin resistance in *Staphylococcus aureus*. J Clin Microbiol. (1999) 37:1591–4. 10.1128/JCM.37.5.1591-1594.1999 10203531 PMC84841

[36] Sakoulas G Gold HS Venkataraman L Degirolami PC Eliopoulos GM Qian Q. Methicillin-resistant *Staphylococcus aureus*: Comparison of susceptibility testing methods and analysis of mecA-positive susceptible strains. J Clin Microbiol. (2001) 39:3946–51. 10.1128/JCM.39.11.3946-3951.2001 11682512 PMC88469

