# Supplementary material for: Immunofluorescence Targeting PBP2a Protein: A New Potential Methicillin Resistance Screening Test
Source: Front Vet Sci. 2021 Nov 30;8:740934. doi: 10.3389/fvets.2021.740934 (PMC8669817; doi:10.3389/fvets.2021.740934)
Supplement: Supplementary file 1 [file Data_Sheet_1.pdf]

Supplementary Material

1 Supplementary Tables

Supplemental Table 1. The table shows the results of the immunofluorescence assay replicates and a short description of the immunolabeling. All immunolabeled cell walls were marked intensely.

Isolate	IF_1	IF_2	IF_{DEF}	description
Pos ctr	+	+	+	numerous bacteria showed a clear cell wall positivity, while the majority of them are negative
Neg ctr	-	-	-	no bacteria stained positive
SP01	-	-	-	no bacteria stained positive
SP02	-	-	-	no bacteria stained positive
SP03	-	-	-	no bacteria stained positive
SP04	+	+	+	numerous bacteria showed a clear cell wall positivity, while the majority of them are negative
SP05	-	+	+	a few clusters of bacteria showed a clear cell wall positivity, while the majority of them are negative
SP06	+	+	+	the majority of bacteria showed a clear cell wall positivity, while a minor number of them are negative
SP07	+	+	+	the majority of bacteria showed a clear cell wall positivity, while a minor number of them are negative
SP08	-	-	-	no bacteria stained positive
SP09	+	+	+	numerous bacteria showed a clear cell wall positivity, while the majority of them are negative
SP10	-	-	-	no bacteria stained positive
SP11	-	-	-	no bacteria stained positive
SP12	+	+	+	the majority of bacteria showed a clear cell wall positivity, while a minor number of them are negative
SP13	-	-	-	no bacteria stained positive
SP14	-	-	-	no bacteria stained positive
SP15	-	-	-	no bacteria stained positive
SP16	-	-	-	no bacteria stained positive

SP17	+	+	+	the majority of bacteria showed a clear cell wall positivity, while a minor number of them are negative
SP18	-	-	-	no bacteria stained positive
SP19	-	-	-	no bacteria stained positive
SP20	-	-	-	no bacteria stained positive
SP21	+	-	+	rare bacteria showed a clear cell wall positivity, while the majority of them are negative
SP22	-	-	-	no bacteria stained positive
SP23	-	-	-	no bacteria stained positive
SP24	-	-	-	no bacteria stained positive

IF_1: first immunofluorescence replicate

IF_2: second immunofluorescence replicate

IF_{DEF}: results of immunofluorescence after 2 replicates

Pos ctr: positive control

Neg ctr: negative control

2 Supplementary Figures

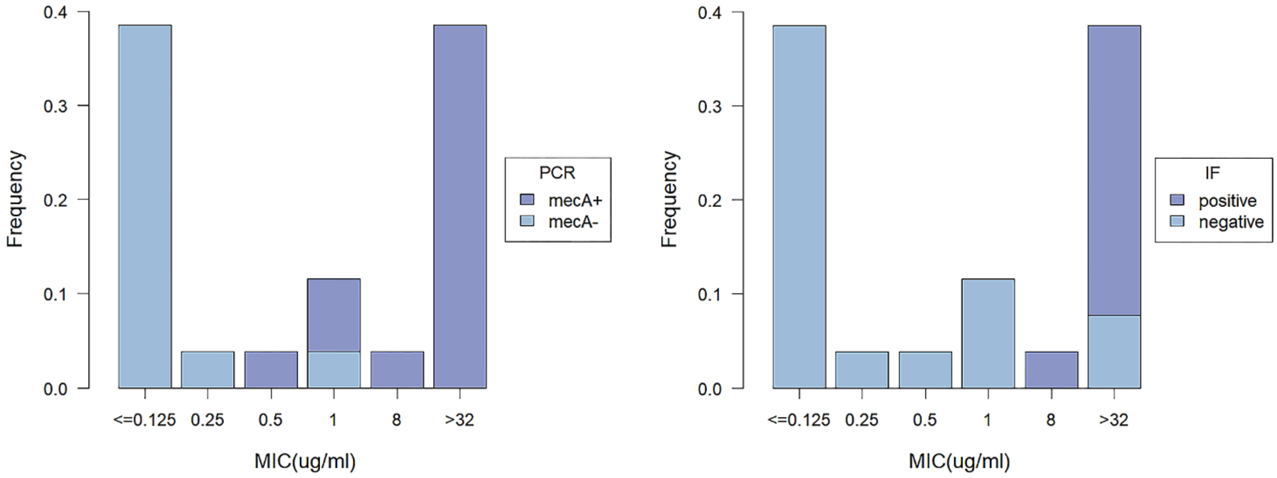

Supplementary Figure 1. MIC values compared to PCR and immunofluorescence targeting PBP2a protein in SP isolates. **(A)** Proportion of *mecA*+ and *mecA*- SP tested by PCR at each MIC value. **(B)** Proportion of cases with PBP2a expression detected and not detected by immunofluorescence assay at each MIC value.
